# Early and Mid-Term Implications of the COVID-19 Pandemic on the Physical, Behavioral and Mental Health of Healthcare Professionals: The CoPE-HCP Study Protocol

**DOI:** 10.3389/fpsyg.2021.616280

**Published:** 2021-02-02

**Authors:** Mohammed Y. Khanji, Carmela Maniero, Sher NG, Imrana Siddiqui, Jaya Gupta, Louise Crosby, Sotiris Antoniou, Rehan Khan, Vikas Kapil, Ajay Gupta

**Affiliations:** ^1^Barts Heart Centre, St. Bartholomew’s Hospital, Barts Health NHS Trust, London, United Kingdom; ^2^Newham University Hospital, Barts Health NHS Trust, London, United Kingdom; ^3^William Harvey Research Institute, Queen Mary University London, London, United Kingdom; ^4^Woodgrange Medical Practice, London, United Kingdom; ^5^Wellbeing Hub, Newham Training Hub, London, United Kingdom; ^6^Waltham Forest and East London (WEL) CCGs, London, United Kingdom; ^7^Barnet, Enfield and Haringey Mental Health Trust, London, United Kingdom

**Keywords:** COVID-19, healthcare professional, wellbeing, mental health, burnout, anxiety, depression, pandemic

## Abstract

**Introduction:**

The COVID-19 pandemic has led to unprecedented strain to healthcare systems worldwide and posed unique challenges to the healthcare professionals (HCPs) and the general public.

**Objectives:**

The aim of this study is to evaluate the impact of COVID-19 on the mental health, behavioral, and physical wellbeing of HCPs in the early and mid-term periods of the pandemic in comparison to non-HCPs. Thus, facilitating and guiding optimum planning and delivery of support to HCPs.

**Methods and Analysis:**

An observational cross-sectional survey and cohort study aiming to enroll over 1050 participants (minimum, 800 HCPs and 250 controls). Study questionnaires will be completed at baseline and after 6-weeks and 4-months. Recruitment initiated July 2020. The study was designed in London, United Kingdom, but open to participants worldwide. Baseline: Questionnaires comprising of validated self-administered screening tools for depression, anxiety, sleep-related issues, wellbeing, and burnout. The questionnaires also explore changes in behavior and physical wellbeing of the participants. In addition, associations of these mental health and behavioral factors with work-related factors and support will be explored. Six-weeks and 4-months follow-up: Follow-up questionnaires will assess change in symptoms of anxiety and depression, sleep disorders, use of alcohol and other substances, behavioral or interpersonal relationship changes. Physical wellbeing will be assessed through the presence of suspected or confirmed COVID-19 infection and absence from work. We will also evaluate the impact of variable provision of personal protection equipment (supply and training), extended working hours, and concern for the wellbeing of family members, anxiety levels, and evidence of burnout.

**Statistical Considerations:**

The study has 80% power to detect a 10% difference of combined depression and/or anxiety symptoms between the groups using two-sided type 1 error at 0.05 at baseline. Assuming that only 50% of these HCPs agree to be a part of a cohort survey, we will have 80% power to detect around 12% difference in the two groups in reported physical symptoms (20% vs. 32.3%), or prevalence of depression and/or anxiety at the end of the study.

**Ethics:**

The study was approved by the Cambridge East, Research Ethics Committee (20/EE/0166).

**Trial Registration Number:**

ClinicalTrials.gov, NCT04433260.

## Introduction

The United Kingdom and the rest of the world now face a pandemic caused by a novel coronavirus, severe acute respiratory syndrome coronavirus 2 (SARS-CoV2). At the time of writing 1st October 2020) there were more than 34 million confirmed cases worldwide with over 1,000,000 deaths^1^. Healthcare professionals (HCPs) are at higher risk of developing life-threatening infectious diseases through exposure to respiratory droplets, aerosols, and contact with patients’ blood or body fluids. This has also been demonstrated in previous epidemics such as the Ebola virus disease in 2014 and Severe Acute Respiratory Syndrome (SARS) a decade earlier, which were associated with very high fatality rates in HCPs (Styra et al., 2008; Forrester et al., 2014; Alfaraj et al., 2018). Whilst efforts to minimize the physical impact of infectious outbreaks take precedence, the potential mental health impact of such pandemics in the short-term and beyond should not be neglected (Barello et al., 2020; Galli et al., 2020; Siddiqui et al., 2021).

Previous studies conducted on the mental health impact of infectious outbreaks have found significant burden among healthcare workers and the general public. During the SARS outbreak, healthcare workers in a Beijing hospital who were quarantined, worked in a high-risk clinical setting or had family or friends infected with SARS, reported substantially more post-traumatic stress symptoms compared to those without (Xiang et al., 2020).

Increased exposure and unprecedented large-scale quarantine measures have a negative mental health impact on the public, in addition to the already tangible economic repercussions (Thompson, 2020; Weiss and Murdoch, 2020). Increased workload alongside a suboptimal working environment of inadequate personal protection equipment (PPE), risk of nosocomial transmission and constant changes in work structure can have detrimental effects on the mental wellbeing of HCPs. The need to isolate for fear of infecting friends and relatives results in loss of a social support network, further compromising the psychological resilience of HCPs.

Several studies exploring mental health impact of the current COVID-19 pandemic and risk factors for this have since been performed (Kisely et al., 2020). Whilst some individual studies have suggested an increased anxiety and risk of mental health problems in HCPs compared to non-HCPs (Zhang et al., 2020), subsequent meta-analyses have found a similar prevalence of anxiety and depression between healthcare workers and healthy controls from the general public (Krishnamoorthy et al., 2020; Luo et al., 2020; Pappa et al., 2020).

This observation can, in part, be explained by the different roles of HCPs. A study by Lu et al. (2020) demonstrated higher levels of psychological distress in HCPs working in hospital, compared to administrative staff. Work-related risk factors including close contact with infected patients, level of work experience and organizational support provided have also been shown to impact the psychological effect of emerging virus outbreaks (Kisely et al., 2020). These effects may be more prominent in junior or trainee doctors likely due to having to work in unfamiliar environment, with disrupted training and variable supervision (Kisely et al., 2020).

Healthcare professionals work under different schedules, including regular office hours, shift work, and swing shifts. Shift work and stressful work-related situations have been linked to poor mental health (Torquati et al., 2019). There is an established body of literature that has demonstrated the prevalence among physicians of a range of sleep-related issues, substance use and mental health disorders (Mihailescu and Neiterman, 2019; Petrie et al., 2019). These problems increase the risk of burnout or errors on the job. In those with pre-existing mental health disorders, there may be increased psychosocial problems, an increased risk of suicidal behavior, increased alcohol, or other psychoactive substance use, or a more severe form of the viral illness (Possamai, 2007; Greenberg et al., 2020).

Of note, most of these studies have evaluated only the immediate psychological impact of COVID-19 on HCPs and the general public. Moving forward, it is vital we identify the at-risk population and pre-disposing factors to higher psychological distress in order to design and target effective interventions to minimize the mental health impact of COVID-19. In the CoPE-HCP study, we aim to study the early and mid-term impact on mental and physical wellbeing in different cohorts of HCPs compared to the general population.

## Study Objectives

### Primary Objective

(1) To evaluate the prevalence of symptoms of anxiety and depression at the time of COVID-19 pandemic amongst HCPs in direct patient-facing roles, as compared to colleagues/participants in non-patient facing roles.
(2) To determine the change in symptoms of anxiety and depression during the follow-up period amongst HCPs in direct patient-facing roles, as compared to colleagues/participants in non-patient facing roles.
(3) To evaluate the change in proportion of those with suspected or confirmed COVID-19 during follow-up period amongst HCPs in direct patient-facing roles, as compared to colleagues/participants in non-patient facing roles.

### Secondary Objective

(1) To assess the impact of COVID-19 pandemic on symptoms of anxiety and depression and behavioral changes in HCPs in comparison to the non-HCPs at baseline and follow-up.
(2) To assess the impact of COVID-19 related symptoms of anxiety and depression and behavioral changes on the subsequent physical wellbeing and absence from work due to illness.
(3) To study the relationship of perceived stressors, such as PPE provision, work hours, future vaccine introduction, staffing levels and support at work, on the physical and mental health of HCPs.
(4) To compare the differences in the prevalence of symptoms of anxiety and depression and behavioral changes, if any, by role and years of experience of HCPs.
(5) To assess the impact of COVID-19 pandemic on the symptoms of anxiety and depression and behavioral changes of HCPs in the United Kingdom compared with HCPs in Europe, Asia, Africa, America, and Australasia.

## Methodology

### Study Design

**FIGURE 1:**
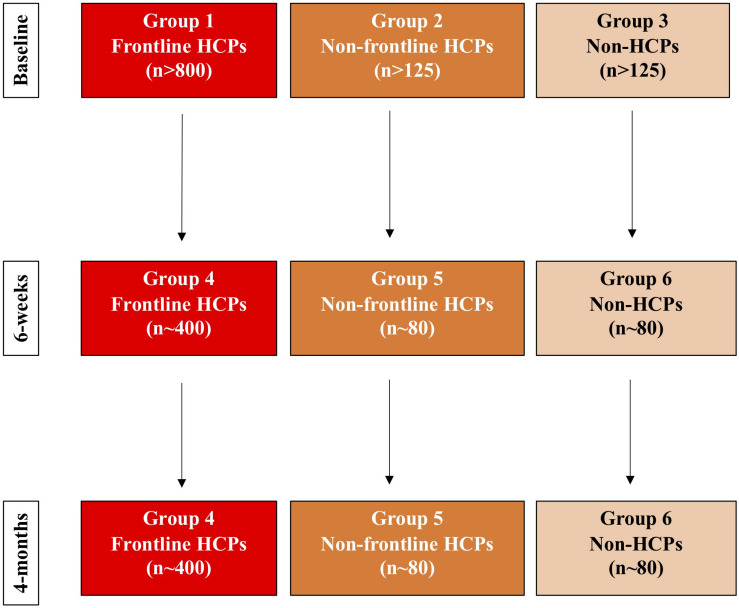
Study protocol flow diagram.

An observational cross-sectional survey and cohort study design. A minimum of 1050 participants will be enrolled (minimum of 800 HCPs and 250 controls). Figure 1 shows the summary of the study scheme. The study recruitment was initiated on the 24th July 2020, just after the first peak of the pandemic (particularly in the United Kingdom and Western Europe), following formal ethical approval. The study is conducted as an online survey and can be complete by participants globally (see Supplementary Data Sheet 1 for questionnaire). The study is designed in London, United Kingdom and we envisage that as a consequence a large proportion of the participants will be from this region.

### Participant Selection

This is an international multicentric study enrolling three groups of participants.

#### Group 1

Healthcare professionals in direct contact with patients confirmed or suspected as having COVID-19 (*n* > 800).

Participants providing consent will be recruited for the follow-up questionnaire study (Group 4).

#### Group 2

Healthcare professionals in non-patient facing roles, not directly in contact with patients confirmed or suspected as having COVID-19 (*n* = 125, internal HCP control).

Participants providing consent will be recruited for the follow-up questionnaire study (Group 5).

#### Group 3

Non-Healthcare academic and research staff of Queen Mary University of London, and other professionals not working with patients confirmed or suspected as having COVID-19 (*n* = 125, population control).

Participants providing consent will be recruited for the follow-up questionnaire study (Group 6).

### Inclusion Criteria

(1) Aged ≥18 years
(2) Electronic consent given
(3) Belonging to one to the following groups:
  (a) HCPs with direct patient facing roles
  (b) Healthcare staff with no direct patient contact
  (c) Non-healthcare academic staff with no direct patient contact

### Exclusion Criteria

(1) Those who are not able to understand written English will be excluded by the design and methodology of the study, as the study invitation and all other information is provided in English.

#### Study Time Points

The study questionnaire will be conducted at baseline, after 6-weeks and 4-months for follow-up. The COVID pandemic in the United Kingdom started in March 2020 and the initial survey therefore assesses the early phase (baseline) and the follow-up (after 6-weeks and 4-months) questionnaires assess the mid-term impact.

##### Baseline

Recruitment will be open for approximately 6-weeks, starting from launch study date (24th July 2020).

##### Follow-up study

All participants consenting for follow-up will be sent further questionnaires after 6-weeks (up to 8-weeks) and 4-months (up to 6-months), from date of completion of the baseline questionnaire, to assess for change from baseline. To improve the uptake, we will also send weekly reminders (no more than 3) to those who have not completed the survey at first request. We expect that about 60% from each of the three baseline groups will agree to take part in the follow-up study, and about two-third of those will respond to the follow-up surveys.

### Endpoints

#### Primary Endpoints

(1) Prevalence of anxiety and/or depression at baseline.
(2) Change in prevalence of combined anxiety and depression from baseline.
(3) Change in proportion of those who report signs and symptoms, or evidence consistent with COVID-19 from baseline to the end of study.

#### Secondary Endpoints:

(1) Prevalence of combined anxiety, depression, or sleep disorder at baseline.
(2) Prevalence of those with sleep disorders at baseline.
(3) Change in prevalence of anxiety, depression, and sleep disorder from baseline to the two follow-up time points.
(4) Change in prevalence of burnout from baseline to the two follow-up time points.
(5) Proportion of those with low mental wellbeing at baseline and follow-up.
(6) Change in behavioral habits such as smoking and alcohol intake from baseline to follow-up.
(7) Proportion of those who report suspected or confirmed diagnosis of COVID-19.
(8) Proportion of those who report their working conditions adversely affecting their personal relationships.

## Definitions of Various Endpoints: How We Will Assess Them

### Psychological

#### Presence of Anxiety

The presence of anxiety is screened using the Generalized Anxiety Disorder-7 (GAD-7) assessment. This is a validated self-administered patient questionnaire used as a screening tool and severity measure for generalized anxiety disorder (GAD). The minimum score is 0 and maximum score 21. The following scoring system will be employed (Kroenke et al., 2001):

*Mild: 5–9; Moderate: 10–14; Severe: >15*.

A score of ≥10 has a sensitivity of 89% and specificity of 82% for GAD. The GAD-7 scoring tool has also been shown to have acceptable sensitivity and specificity for other types of anxiety disorders such as panic disorder, social phobia, and post-traumatic stress disorder (sensitivity 68% and specificity 88% with a cut off score of 10, for any anxiety disorder).

#### Presence of Depression

The presence of depression is screened using the Patient Health Questionnaire-9 (PHQ-9). This is a validated nine-item questionnaire designed to screen for depression, often used in a primary care setting. A PHQ-9 score of ≥10 has an 88% sensitivity and specificity for major depression (Spitzer et al., 2006). The severity of depression is rated as follows:

None: 0–4; Mild: 5–9; Moderate: 10–14; Moderately severe: 15–19; Severe: 20–27.

#### Sleep-Related Issues

Sleep related issues are assessed through the Insomnia Sleep Index (ISI). This is a validated seven-item self-report questionnaire assessing the nature, severity and impact of insomnia, evaluating aspects such as severity of sleep onset, sleep maintenance, sleep dissatisfaction, and interference of sleep difficulties (Tennant et al., 2007). The score categories are as follows (Bastien et al., 2001):

0–7: No clinically significant insomnia; 8–14: Sub threshold insomnia; 15–21: Clinical; insomnia (moderate severity); 22–28: Clinical insomnia (severe).

#### “Mental Wellbeing”

Mental wellbeing is assessed using the Short Warwick-Edinburgh Mental Wellbeing Scale (SWEMWBS). This has been validated for use in the general population and facilitates monitoring mental wellbeing in the general population (Tennant et al., 2007):

Scores of 7–17 suggest probable depression or anxiety; Scores of 18–20 suggest possible depression or anxiety. Scores range from 7 to 35. Higher scores indicate higher positive mental wellbeing.

#### Burnout

Burnout was assessed using single Item measures for Emotional Exhaustion and Depersonalization (West et al., 2009). This 2-Question summative score has been shown to be correlated with two items from the Maslach Burnout Inventory (Li-Sauerwine et al., 2020).

### Lifestyle and Physical Health

#### Behavioral Habits, Such as Smoking, Alcohol Intake and Recreational Drug Use

Self-reported measures through responses to customized questions developed the research team (see Supplementary Data Sheet 1).

#### Diet and Physical Activity

Customized questions on diet, exercise levels and de-stressing activity.

#### Physical Health

##### Evidence consistent with probable diagnosis of COVID-19

This is self-reported and is assessed through questions regarding the presence of symptoms with the presence of either a self-reported positive test, or self-isolation for 7 days or more.

Customized questions on symptoms of COVID-19, swab and antibody status and days of absence/sick leave taken and potential need for hospitalization.

### Relational and Support

#### Social/Relational

Customized questions on living arrangements and impact of the pandemic on personal relationship.

#### Concerns Related to Workplace and Support

Customized questions developed by the research team to assess self-reported responses to workplace related concerns including availability of support at work.

## Study Questionnaire and Dissemination

This will be achieved through a wide distribution of the electronic survey to HCPs and non-HCP controls. We will seek endorsement and support from professional societies and associations in the United Kingdom and other parts of the world to disseminate this widely. Distribution networks that will be considered include network email distribution lists and relevant social media platforms.

We expect to have a larger distribution of the questionnaire in the United Kingdom, but we are aiming at achieving an international cohort of participants. Within the United Kingdom, dissemination will be through different NHS Trusts, geographically distinct deaneries involving with overseeing medical training, scientific and medical societies and universities. On an international level we will involve international medical and allied health scientific societies and associations.

There will be an invitation to join the study including an explanation of the reasons of the survey. Any participant taking part in the survey based on the brief description of the study will be deemed to have consented for the study, and no other consent will be required. This cross-sectional survey will include basic information about the participant including demographics, living circumstances, education level and pre-existing physical and mental health conditions. It will also include questions regarding work experience and profession, work circumstances and exposure to COVID-19. Table 1 provides a summary of the aspects assessed and the tools used.

**TABLE 1:** Study variables and respective assessment tools.

Variables	Assessment Tool
***Psychological***	
1. Anxiety	Generalized Anxiety Disorder-7 (GAD-7)
2. Depression	Patient Health Questionnaire-9 (PHQ-9)
3. Sleep-related issues	Insomnia Sleep Index (ISI)
4. “Mental wellbeing”	Short Warwick-Edinburgh Mental Wellbeing Scale (SWEMWBS)
5. Burnout	Abbreviated 2-Question Summative Score
***Lifestyle and physical health***	
6. Behavioral habits (smoking, alcohol intake and recreational drug use)	Customized questions on cigarette smoking and vaping status, alcohol and recreational drug use.
7. Diet and physical activity	Customized questions on diet, exercise levels and de-stressing activity
8. Physical health	Customized questions on symptoms of COVID-19, swab and antibody status and days of absence/sick leave taken or potential need for hospitalization
***Relational and support***	
9. Social/relational	Customized questions on living arrangements and personal relationship
10. Concerns related to work place and support	Customized questions regarding concerns related to workplace and available work-based support

Comparing Groups 1 and 2 with Group 3 allows us to study the effect of a high-risk working environment alone, whether in a patient-facing (Group 1) or non-patient facing role (Group 2), on physical and psychological health. Group 3 may also function as a control group for confounders such as education level and living conditions (external control).

We aim to structure each of the questionnaires such that most of these questions can be answered in no more than 20 min. We will try to incorporate strategies such that relevant sections (rather than the whole) survey can be completed in multiple sittings with previous responses being saved.

## Patient and Public Involvement

As the study is aimed at HCPs, hospital workers, and academic staff, we have consulted with a range of different workers from these groups in refining the research questions, designing the survey and planning the follow-up.

### Procedure for Collecting Data

Data will be collected directly from the web-based survey platform^2^ using pre-defined questions using a combination of Likert scale, one of many tick options and free text etc. Participants will be free to withdraw (actively or by ceasing to complete any questionnaires at any time). Data collected up to the point of no further completion, or withdrawal will be kept for data analysis.

## End of Study Definition

The end of study definition is hierarchical based on collection of completed surveys from *n* > 400 from group 4 AND *n* > 80 from group 5 and 6. If not achieved, then at the end of 12 months from study opening.

## Statistical Considerations

### Sample Size

We aim to collect data from minimum of 800 HCPs, and minimum of 125 non-patient facing HCPs and 125 non-HCPs (total sample size of 1050). If minimum of 40% of HCPs report primary outcome (combined either depression and/or anxiety symptoms) compared with a maximum of 30% non-HCPs/non-patient facing HCPs, we will have just of over 80% power to detect significant difference using two-sided type 1 error at 0.05.

Assuming that only 50% of these HCPs agree to be a part of a cohort survey (*n* = 400), we will have at least 40% (*n* = 160) who have reported either depression or anxiety. We will have 80% power to detect around 12% difference in the two groups in reported physical symptoms (20% vs. 32.3%), or prevalence of depression and/or anxiety at the end of the study. We will also have about 80% power at two-sided alpha set at 0.05 to detect difference of 15% between baseline and the end-of study for all the primary and secondary objectives, amongst those who have reported anxiety or depression symptoms at baseline (*n* = 160). In the other arm, we have enough power to detect smaller changes from baseline.

Our assumptions here are based on most conservative estimates. If we are able to recruit more than the minimum numbers, our power will improve substantially, and we will be able to detect smaller differences too.

### Method of Analysis

We will use the STATA 15 statistical software for analysis. Chi-square test will be used to compare the difference in prevalence of anxiety, depression and other variables between the groups at baseline. We will evaluate for changes in proportion of those outcomes at baseline to the end of the study using paired McNemar test. We will use logistic regression to assess the factors at baseline related to development of physical symptoms, overall, and in the HCPs group alone. Data will be described using appropriate descriptive statistics.

We will collect information for potential confounding factors such as age, gender, education level, and health conditions from both HCPs and their controls and adjust for these in the analysis. For all validated tools, we will use appropriate and previously published cut-offs to categorize them. For example, for the primary end point of anxiety, we will use the cut-off related to moderate anxiety, but also do a sensitivity analysis using cut-off using “mild” anxiety definition. Similar and consistent strategy will be followed for other validated tools. We will perform logistic regression for binary outcomes, such as those listed in our primary and secondary outcomes, after adjusting for pre-defined confounders including age, gender, and years of education. The choice of these *a priori* confounder is based on the significant association that exists for most of the study outcomes. We have also adjusted for time since the self-identified peak of the pandemic as this will impact on outcomes such as anxiety and depression. However, for the outcome of developing COVID-19 infection and change in prevalence of COVID-19 infection, we will also adjust for pre-existing medical conditions.

We will also stratify HCPs according to their roles (doctors, nurses, pharmacists, healthcare assistants etc.), and will evaluate and compare the endpoints by respective roles. Comparison between HCPs from the United Kingdom and outside will be undertaken adjusting for the self-identified peak of the pandemic in that region. We also will assess whether the years of experience (as a categorical variable) has any impact on the measured outcomes.

## Ethical Considerations

### Ethical Approval

NHS Research Ethics Committee (REC) approval has been obtained for the study (protocol, consent form, all written material to be provided to the participant and all advertisements that may be used for participant recruitment). Appropriate reports on the progress and any other notifications of this trial by the Investigator will be made to the REC and the Sponsor in accordance with the applicable governance regulations and in agreement with policy established by the Sponsor.

### Risks, Burdens, and Benefits

There are no significant risks or benefits associated with participating in this survey.

There is an ethical concern about what we should do for participants in the cohort survey displaying mental or physical wellbeing concerns. We therefore clarify that our questionnaire remains a screening tool and does not provide final clinical diagnosis of any physical or psychiatric conditions. We emphasize the importance for participants to seek clinical advice from their occupational health department or GP, should they feel the need to. In addition, signposting or links to mental health support websites or services are provided on the survey platform and on our study website. These include (but not exclusive to): occupational health departments at workplace (general practitioner, Health Education England Professional Support Unit, FRONTLINE NHS helpline, MIND, health professional unions etc.).

### Informed Consent

Participant information sheet and consent will be available electronically on the study website. All participants are required to provide informed consent prior to completing the questionnaire.

## Study Limitations

This study has certain limitations. The online survey-based methodology relies on self-reported responses can be subjective. For example, survey responses may provide a one-sided interpretation of events and is dependent on participants’ recall. Non-response to optional questions may also limit data interpretation. There is the potential for selection bias which is inherent in studies with voluntary participation.

Whilst the survey questions attempt to address predicted confounders such as participant demographics, education level and physical and mental health, we acknowledge that there may be unknown confounders, particularly in a study conducted internationally. Although we try to control for this, we acknowledge that by including participants from multiple countries, we will capture responses occurring at different phases of the COVID-19 pandemic. There may also be heterogeneity in responses as a result of different financial and health policies adopted worldwide. We hope to account for some of these differences by collecting basic information regarding the participant’s demographics and characterizing these differences in our analysis.

Many of the limitations are inherent to the online questionnaire-based methodology, but we have chosen to balance the limitations against the benefits, which include easy accessibility and its ability to overcome geographical barriers.

## Discussion

As suggested by recent studies in this area, we expect that the COVID-19 pandemic will have a significant impact on the HCPs working on the frontline, and that this will have significant impact on their physical and mental health over the period of follow-up. Direct patient facing roles are expected to have negative impact on psychological and physical wellbeing, compared to non-patient facing roles or compared to the general public. In particular we expect that frontline HCP workers will have increased levels of anxiety, depression and sleep disorders compared to non-patient facing HCPs and control populations. We also hypothesize that, compared to controls, they will report significant behavioral changes regarding habits such as smoking, diet, and exercise, and that the pandemic will be likely to impact on their personal relationships. We also hypothesize significant changes in levels of burnout.

We surmise the adequate provision of appropriate PPE alongside necessary training will impact levels of anxiety as well as recorded absence from work. This stressful event may also have implications for the early and medium-term mental health of these workers and may also have an impact on their physical wellbeing.

### Relevance of Finding for Clinical Practice/Prevention

The findings of this study will help to outline the impact of the COVID-19 pandemic on HCPs, identify the needs of HCPs and help to improve design and delivery of support systems. The wellbeing of HCPs is vital in order for them to be able to continue providing the vital services during the pandemic and beyond. We plan to share the findings with healthcare leaders, the scientific community and individual staff members to allow better understanding and support structures for maintaining wellbeing.

## Ethics Statement

The studies involving human participants were reviewed and approved by the Cambridge East, Research Ethics Committee (20/EE/0166). The participants will provide their written informed consent to participate in the study via completion of the survey.

## Author Contributions

All authors listed above fulfill all three International Committee of Medical Journal Editors (ICMJE) guidelines for authorship, which are (1) substantial contributions to conception and design, acquisition of data or analysis, and interpretation of data; (2) drafting the article or revising it critically for important intellectual content and (3) final approval of the version to be published. All authors helped in the design of the study, were responsible for editing and providing guidance on the manuscript, critically revising the manuscript, and approved the final version of this protocol document for submission. MK, AG, and CM were responsible for coordinating the contribution of all authors to the manuscript. All were involved in the development of the protocol. MK, AG, CM, SN, and VK were responsible for drafting the manuscript.

## Conflict of Interest

The authors declare that the research was conducted in the absence of any commercial or financial relationships that could be construed as a potential conflict of interest.

## Footnotes

1 https://coronavirus.jhu.edu/

2 www.surveymonkey.com
