# Supplementary material for: Early and Mid-Term Implications of the COVID-19 Pandemic on the Physical, Behavioral and Mental Health of Healthcare Professionals: The CoPE-HCP Study Protocol
Source: Front Psychol. 2021 Feb 2;12:616280. doi: 10.3389/fpsyg.2021.616280 (PMC7884763; doi:10.3389/fpsyg.2021.616280)
Supplement: Supplementary file 1 [file Data_Sheet_1.pdf]

CoPE-HCP Study

COVID-19 disease and Physical & Emotional wellbeing of Healthcare Professionals.

Thank you for your interest in the CoPE-HCP study. We want to assess the physical, emotional and psychological effects of the COVID-19 pandemic on our healthcare professionals. We will collect data from both healthcare and non-healthcare professionals/academics. This will help us understand the differences in the impact of COVID-19 disease in the two groups.

This study is conducted in accordance with the ethical standards of the institutional research ethics committee (REC number 20/EE/0166) and is a registered clinical trial on clinicaltrials.gov (NCT04433260)

It has two distinct phases. You can choose to be part of one or both phases.

In the initial survey (phase 1), you will be asked to complete a set of questions about your current work and living environment, physical and mental health as well as wellbeing. There will also be questions around the support available at your workplace, the provision of personal protective equipment (PPE) and your work pattern. We estimate this to take no longer than 20 minutes. ***You can do them at your own pace and speed, and it doesn't have to be in one sitting.***

In Phase 2 of the study, we aim to reassess your physical and psychological well-being 6 weeks and 4 months following the initial survey. Our objective is to evaluate the changes in behaviour, mood and wellbeing and, whether there were any perceived issues with physical wellbeing.

Further details about the study can be accessed here.

Please read the participant information sheet here.

Please note that we do not ask for any personal data, except for your contact email address (only if you opt in for follow-up surveys in phase 2). Using your email address will allow us to identify your initial response in Phase 1 and link them to your future responses in follow-up surveys.***The surveys are conducted entirely in English. It is at the participant's (your) discretion whether to proceed, assuming you have understood the study information given. All data will be treated confidentially and under data protection act 2018*****.**

* I have read and understood the information provided about the study

☐ Yes

* Are you happy to proceed with this survey?

☐ Yes

Note on how to do this survey:

We understand your time is valuable but would appreciate if each question can be answered as fully as possible, as all information provided will be helpful to us! **You can take a break** when answering the survey and continue the survey from where you left
(i) provided you use the same device to go back to the survey **OR**
(ii) access a unique link sent to your email address (this allows access from a different device)

If you feel you need urgent support, please use urgent helplines such as Samaritans on 116 123 or Mind on 0300 123 3393) or contact your GP or occupational health.

* Are you are happy for us to contact you for the follow-up surveys? *[you can still proceed to the initial survey without providing this]*

- ◯ Yes
- ◯ No
- ◯ Do not know yet

CoPE-HCP Study

* Please provide your email address

[ ]

CoPE-HCP Study

About yourself

This section contains questions regarding basic information about you, your place of living and work, level of education, relationship status and pre-existing physical and mental health conditions. Please tick whichever option is most applicable

* 1a. How did you find out about this study?

- ◯ Email invitation
- ◯ Social media (Twitter, Facebook, WhatsApp, etc.)
- ◯ Institutional/Organisational newsletter
- ◯ Queen Mary University London (QMUL) website
- ◯ Search Engine (Google, Yahoo, etc.)
- ◯ Word of Mouth
- ◯ Other

Other (please specify)

* 1b. Do you work in the UK?

- ◯ UK
- ◯ Rest of the world

CoPE-HCP Study

* 2a. Which region in the UK do you work at?

- ◯ East of England
- ◯ London
- ◯ East Midlands
- ◯ West Midlands
- ◯ North East and Yorkshire
- ◯ North West
- ◯ South East
- ◯ South West
- ◯ Scotland
- ◯ Wales
- ◯ Northern Ireland

CoPE-HCP Study

* 2a (1) If you work in London, please indicate below who your primary employer is:

CoPE-HCP Study

* 2b. In Which region in the world do you work?

- Africa
- Asia
- Australia
- Europe
- North America
- Central America
- South America
- Other (please state):

2b(1) Please state which country you are based in *(optional)*

CoPE-HCP Study

* 3. What is your age group?

- 18-25 years
- 26-35 years
- 36-50 years
- 51-60 years
- 61-70 years
- > 70 years

* 4. What is your ethnicity?

- ◯ White British
- ◯ White – any other background
- ◯ Black African-Caribbean/ African
- ◯ Black – any other background
- ◯ Mixed White and Black
- ◯ Mixed White and Asian
- ◯ Mixed any other / Multiple ethnic background
- ◯ South Asian (Indian/Pakistani/Bangladeshi)
- ◯ Chinese
- ◯ Any other Asian background
- ◯ Any other ethnic group
- ◯ I prefer not to disclose my ethnicity

* 5. Which gender do you identify with?

- ◯ Male
- ◯ Female
- ◯ Prefer to self-define
- ◯ Prefer not to say

* 6. What is your relationship status?

- ◯ Single
- ◯ Divorced
- ◯ Married/Living with partner or family
- ◯ I prefer not to disclose
- ◯ Other (please specify)

____

* 7. Please indicate the number of people living in your household (including yourself)

- ◯ 1
- ◯ 2
- ◯ 3-5
- ◯ 6 or more

* 8. What is your highest level of education?

- ○ GCSE or equivalent (i.e minimum of 10 years of school education)
- ○ A-levels or equivalent (i.e. minimum of 12 years of school education or vocational training)
- ○ Bachelor’s degree or equivalent
- ○ Postgraduate diploma or equivalent
- ○ Master’s degree or equivalent
- ○ PhD
- ○ Other (please specify)

* 9. Are you diagnosed with any of the following physical conditions?

- ☐ Chronic lung disease
- ☐ Compromised immune system (i.e. undergoing chemotherapy, on regular immunosuppression)
- ☐ Heart disease
- ☐ Obesity
- ☐ Diabetes
- ☐ Liver disease
- ☐ Hypertension (high blood pressure)
- ☐ Other underlying health condition that might put you at increased risk of Covid infection
- ☐ None of the above
- ☐ Prefer not to disclose
- ☐ Other

CoPE-HCP Study

* 10. Are you diagnosed with any of the following common mental health conditions?

- [ ] Depression
- [ ] Generalised anxiety disorder
- [ ] Panic disorder
- [ ] Obsessive-compulsive disorder
- [ ] Post-traumatic stress disorder
- [ ] Psychotic disorder
- [ ] None of the above
- [ ] Prefer not to disclose
- [ ] Other

* 11. In your region (i.e. where you work), when was the largest number of COVID-19 cases reported (i.e when was the peak of COVID-19 pandemic)

- ○ February – March 2020 (or, > 4 months ago)
- ○ April - May 2020 (or, 3-4 months ago)
- ○ June 2020 (or, 1-2 months ago)
- ○ July 2020 (upto 1 month back)
- ○ August 2020
- ○ Do not know
- ○ Not applicable

CoPE-HCP Study

About your work

These questions are about your work, either as a health care professional or not

* 1. Do you work full time (>35 hours a week) or part-time?

- ○ Part-time
- ○ Full time

About your work

These questions are about your work, either as a health care professional or not

* 1 (a) If you work part-time, please specify an estimate number of hours you work in a week:

- ◯ <8 hours
- ◯ 8-16 hours
- ◯ 17-24 hours
- ◯ 25-32 hours

* 2. In which sector do you work?

- ◯ Healthcare: Primary care
- ◯ Healthcare: Hospital
- ◯ Healthcare: Community
- ◯ Healthcare: administration/estates/IT etc
- ◯ Non-healthcare sector
- ◯ University
- ◯ Other (please specify)

CoPE-HCP Study

* 1. Are you a healthcare worker? (Healthcare workers are self-defined as those who work in primary, community or hospital settings; either directly or indirectly helping in the patient care services. For example, this may include those who work in hospital or community care settings and provide support for direct patient-care services.)

- ◯ Yes
- ◯ No

CoPE-HCP Study

The following section applies <u>ONLY to health care professionals.</u>

* 1. If you are a healthcare professional, are you in a patient facing/caring role (i.e. you routinely come in contact with patients).

- Yes
- No
- Not applicable

* 2. If you are a healthcare professional, what is your role?

- Senior Doctor (consultant or GP or staff specialist)
- Higher specialist trainee (post core-training, fellowship etc)
- Clinical fellow or other junior doctor
- Core trainee (medicine, surgery, psychiatry or GP trainee)
- Foundation doctor (first two years after medical school)
- Nurse or Midwife
- Healthcare assistant
- Allied Healthcare professional (*physiotherapist, occupational therapist, radiographer etc*)
- Pharmacist
- Other patient facing HCW (phlebotomists, cleaners, porters)
- Non-patient facing HCW (e.g. laboratory technicians, administrative staff)
- Not applicable

* 3. You have spent most time working in which of the following areas over the past three months?

- Hospital - Accident and Emergency/Intensive Care
- Hospital – Acute Medicine/Other medical specialities
- Hospital- Surgical specialities
- Hospital – Elective outpatient services/procedures
- Hospital – Mental Health
- Hospital – In reach speciality services
- GP surgery
- Community mental health
- Community in-patient or minor-injury/walk-in services (including hospice/rehabilitation units)
- None of the above
- Not applicable

* 4. How many full-time years of clinical experience do you have, after your basic healthcare training (e.g. medical, nursing, physiotherapy training)?

- <3
- 3-8
- 9-14
- 15-20
- > 20 years
- Not applicable

* 5. At the peak of the COVID-19 pandemic, how many hours per week would you have worked on average (including paid and unpaid hours)?

- <35 hours/week
- 35-45 hours
- > 45 hours
- Not applicable

* 6. Have you been redeployed to a different team/speciality as a result of the COVID-19 pandemic?

- Yes
- No
- Not applicable

* 7. If you have been redeployed, did you receive adequate training before starting in your new post?

- Yes
- No
- Not applicable

* 8. How often did you feel your team was understaffed?

- Never
- 25% of the time
- Half the time
- 75% of the time
- All the time
- Not applicable

* 9. Was personal protective equipment (PPE) adequately provided to staff at your workplace?

- Yes
- No
- Not applicable

* 10. If you worked in patient-facing roles, do you think you received appropriate training for using PPEs?

- Yes
- No
- Not applicable

* 11. Do you consider work-related practices (e.g. working hours, PPE provision, staffing levels) a source of stress/anxiety?

- Yes
- No
- Not applicable

CoPE-HCP Study

COVID-19: Impact on yourself, your workplace and other social aspects

Please answer the following questions, taking into account the changes that may have occurred in your work place and living conditions during the COVID-19 pandemic.

* 1. Is your workplace adhering to local guidelines on social distancing, working from home, reduced personal contacts, personal protective equipment, personal hygiene etc?

- Yes
- No
- Partially
- Prefer not to disclose

* 2. During the COVID-19 pandemic, have you had to make new living arrangements because of friends/family members who are at moderate or high clinical risk?

- Yes
- No
- Prefer not to disclose
- Not applicable

* 3. Are your working conditions adversely affecting your personal relationships?

- Yes
- No
- Prefer not to disclose
- Not applicable

CoPE-HCP Study

COVID-19 and your anxiety levels before, during and at this time

* 4. During the **peak of the COVID pandemic**, please indicate how strongly each statement below is representative of your feelings at that time:

I worried/worry that:

	Strongly Disagree	Disagree	Neutral	Agree	Strongly Agree
My family members may get infected with COVID-19					
COVID-19 will significantly impact my community					
COVID-19 is increasing my general stress levels					
COVID-19 is increasing my financial stress levels					
COVID-19 is increasing my stress about food supplies					

5. Please indicate your anxiety levels in the following stages **(before, at the peak and at present)** of the COVID-19 pandemic:

* 5 (a) How would you rate your anxiety level about your own health, your job, and your families' wellbeing **prior** to COVID-19 pandemic (scale 1-10, 1-zero anxiety and 10 very anxious)?

1 10

* 5 (b)How would you rate your anxiety level about your own health, your job, and your families' wellbeing at the **peak** of the COVID-19 pandemic (scale 1-10, 1-zero anxiety and 10 very anxious)?

1 10

* 5 (c) How would you rate your anxiety level about your own health, your job, and your families' wellbeing **at this point in time** of the COVID-19 pandemic (scale 1-10, 1-zero anxiety and 10 very anxious)?

1 10

CoPE-HCP Study

COVID19 pandemic, physical health and employer's support

* 1. Have you had symptoms (e.g. fever, persistent cough, loss, or change, in your normal sense of taste or smell) suggestive of COVID-19 infection in the last few months?

- Yes
- No
- Prefer not to disclose

* 2. Have you been tested (viral swab or antibody test) for COVID-19?

- Yes – positive swab result, no antibody test
- Yes – positive swab result, positive antibody test
- Yes - positive swab result, negative antibody test
- Yes - negative swab result, no antibody test
- Yes - negative swab result, positive antibody test
- Yes - negative swab result, negative antibody test
- Yes: no swab test, positive antibody test
- Yes: no swab test, negative antibody test
- No: no swab test, no antibody test

* 3. How many days were you off work because you had symptoms suspicious of COVID-19?

- None – 0 days
- 1-7 days
- 8-14 days
- >14 days

* 4. With regards to physical activity, which of the following is true about yourself during the COVID-19 pandemic?

- I am physically less active
- I am as physically active as I was before
- I am more physically active
- I don't know/I'm not sure
- Prefer not to disclose

* 5. With regards to your weight or dress/waist size, which of the following is true during the COVID-19 pandemic?

- There has been no change in my weight, dress or waist size
- My weight, dress or waist size has increased
- My weight, dress or waist size has decreased
- I don't know/I'm not sure
- Prefer not to disclose

* 6. Do you think you received adequate support directly from your supervisors/line managers/direct employers? (Mark on scale, with 1 -as no support and 10 as full and professional support)

1 10

6a. What support did you find most helpful or would have wanted if possible? (please specify what kind of support and provided by whom) - optional

7. If you feel you were supported, please specify which source of support you found helpful?

- [ ] From direct supervisor/line-manager/immediate management
- [ ] Peer groups/fellow workers/fellow professionals
- [ ] Friends and family
- [ ] Employer or company/hospital wide policies
- [ ] Media
- [ ] Societies/other organisations
- [ ] Government policies
- [ ] None of the above
- [ ] Other (please specify)

8. What support did you find most helpful or would have wanted if possible? (please specify what kind of support and provided by whom)? - optional

CoPE-HCP Study

Mood related:

In this section we are requesting you to reflect on your mood during the last two weeks and answer the following:

* 1. Over the last two weeks, how often have you been bothered by any of the following problems?

	Not at all	Several days	More than half the time	Nearly every day
Little interest or pleasure in doing things	◯	◯	◯	◯
Feeling down, depressed, or hopeless	◯	◯	◯	◯
Trouble falling or staying asleep, or sleeping too much	◯	◯	◯	◯
Feeling tired or having little energy	◯	◯	◯	◯
Poor appetite or overeating	◯	◯	◯	◯
Feeling bad about yourself — or that you are a failure or have let yourself or your family down	◯	◯	◯	◯
Trouble concentrating on things, such as reading the newspaper or watching television	◯	◯	◯	◯
Moving or speaking so slowly that other people could have noticed? Or the opposite; being so fidgety or restless that you have been moving around a lot more than usual	◯	◯	◯	◯
Thoughts that you would be better off dead or of hurting yourself in someway	◯	◯	◯	◯

Anxiety related

* 2. Over the **last two weeks**, how often have you been bothered by any of the following problems?

	Not at all	Several days	More than half the time	Nearly every day
Feeling nervous, anxious or on edge	○	○	○	○
Not being able to stop or control worrying	○	○	○	○
Worrying too much about different things	○	○	○	○
Trouble relaxing	○	○	○	○
Being so restless that it is hard to sit still	○	○	○	○
Becoming easily annoyed or irritable	○	○	○	○
Feeling afraid as if something awful might happen	○	○	○	○

CoPE-HCP Study

Sleep related

* 3. Please rate the CURRENT (i.e. **LAST 2 WEEKS**) SEVERITY of your sleep-related issues

	None	Mild	Moderate	Severe	Very Severe
Difficulty falling asleep	○	○	○	○	○
Difficulty staying asleep	○	○	○	○	○
Waking up too early	○	○	○	○	○

* 3.1. How SATISFIED/DISSATISFIED are you with your CURRENT sleep pattern?

- ○ Very Satisfied
- ○ Satisfied
- ○ Moderately satisfied
- ○ Dissatisfied
- ○ Very Dissatisfied

* 3.2 How NOTICEABLE to others do you think your sleep problem is in terms of impairing the quality of your life?

- Not at all Noticeable
- A little
- Somewhat
- Much
- Very much noticeable

* 3.3 How WORRIED/DISTRESSED are you about your CURRENT sleep problem?

- Not at all worried
- A little
- Somewhat
- Much
- Very much worried

* 3.4. To what extent do you consider your sleep problem to INTERFERE with your daily functioning (e.g. daytime fatigue, mood, ability to function at work/daily chores, concentration, memory, mood, etc.) CURRENTLY?

- Not at all interfering
- A little
- Somewhat
- Much
- Very much interfering

CoPE-HCP Study

Behaviour related

* 4.1 Has there been a change in your **smoking** status ***since the start of COVID pandemic***?

- Never smoked
- Already stopped before the start of COVID pandemic
- Smoking less than before the start of COVID pandemic
- Smoking more than before since the start of COVID pandemic
- Smoking same as before the start of COVID pandemic
- Prefer not to say

* 4.2 Has there been a change in your **vaping** status ***since the start of COVID pandemic***?

- Never vaped
- Already stopped vaping before the start of COVID pandemic
- Vaping less than before since the start of COVID pandemic
- Vaping more than before since the start of COVID pandemic
- Vaping same as before the start of COVID pandemic
- Prefer not to say

* 4.3 Has there been a change in your levels of **alcohol** consumption ***since the start of COVID pandemic***?

- Never drank
- Already stopped before the start of COVID pandemic
- Drinking less than before since the start of COVID pandemic
- Drinking more than before since the start of COVID pandemic
- Drinking same as before the start of COVID pandemic
- Prefer not to say

* 4.4 Has there been a change in your levels of recreational drug consumption ***since the start of COVID pandemic***?

- Never taken **recreational drugs**
- Already stopped before the start of COVID pandemic
- Taking less **recreational drugs** since the start of COVID pandemic
- Taking more **recreational drugs** since the start of COVID pandemic
- Taking same as before the start of COVID pandemic
- Prefer not to say

CoPE-HCP Study

Concerns related: during the **peak of** COVID pandemic

Please answer the questions below about your worries during the peak of the COVID pandemic. If your country has not gone through the peak yet, please answer these as your potential worries about the pandemic

* 5. Do you worry about your health as a result of COVID-19 pandemic? Specify on Likert scale of 1-5

	Always	Often	Sometimes	Rarely	Never
Do/did you worry about your health as a result of Covid-19 pandemic?	◯	◯	◯	◯	◯
Do/did you worry about being at greater risk due to not having adequate PPE?	◯	◯	◯	◯	◯
Do/did you worry about your family catching Covid 19 due to your work?	◯	◯	◯	◯	◯
Do/did you worry about not having adequate training to deal with Covid 19 related jobs in workplace?	◯	◯	◯	◯	◯
Do/did you worry about not having adequate supervision in workplace?	◯	◯	◯	◯	◯
Do/did you worry about being redeployed?	◯	◯	◯	◯	◯

CoPE-HCP Study

COVID pandemic: health and lifestyle

* 6.1 Are you managing to eat a healthy diet (with adequate fresh fruits and vegetables)

◯ I was never a healthy eater

◯ I am eating the same as before

◯ I am able to eat healthy meals

◯ I try but do not always manage to eat a healthy diet

◯ I cannot manage to eat a healthy diet

* 6.2 Are you managing to eat regular meals?

- I could never eat regular meals
- My meal frequency is the same as before
- I am able to eat regular meals
- I try but do not always manage to eat regular meals
- I cannot manage to eat regular meals

* 6.3 How have your levels of exercise changed since the start of COVID pandemic

- I never exercised regularly
- I am exercising the same as before
- I am able to exercise more than before
- I try but do not always manage to exercise as before
- I cannot exercise same as before

* 6.4 How have your levels de-stressing activity changed the start of COVID pandemic

- I am doing this the same as before
- I am able to do de-stressing activity more than before
- I try but do not always manage to do de-stressing activities as before
- I cannot do de-stressing activities same as before

CoPE-HCP Study

Below are some statements about feelings and thoughts.

* 7. Please tick the box that best describes your experience of each **over the last 2 weeks**

	None of the time	Rarely	Some of the time	Often	All of the time
I’ve been feeling optimistic about the future	◯	◯	◯	◯	◯
I’ve been feeling useful	◯	◯	◯	◯	◯
I’ve been feeling relaxed	◯	◯	◯	◯	◯
I’ve been dealing with problems well	◯	◯	◯	◯	◯
I’ve been thinking clearly	◯	◯	◯	◯	◯
I’ve been feeling close to other people	◯	◯	◯	◯	◯
I’ve been able to make up my own mind about things	◯	◯	◯	◯	◯

CoPE-HCP Study

8. Please indicate in the box below if there is anything else significantly impacting your mental health and wellbeing during the COVID-19 pandemic that has not been asked about in this survey. *(Optional)*

CoPE-HCP Study

COVID19 and my feeling regarding work

1. Please answer in the likert scale to what degree the following statement are applicable to you for your feeling towards your work.

* Likert Scale responses as 0 = never, and 6 as every day

	Never	A few times a year or less	Once a month or less	A few times a month	Once a week	A few times a week	Every day
I feel burned out from my work	◯	◯	◯	◯	◯	◯	◯
I have become more callous towards people since I took this job	◯	◯	◯	◯	◯	◯	◯

* 2. Do you feel you are more irritable or impatient with your colleagues/patients during the course of your work?

- Yes
- No
- To some extent
- Prefer not to disclose

CoPE-HCP Study

Further information

Thank you for taking the time to complete this survey.

We know these are challenging times and some may find this unchartered territory overwhelming. If you have been affected by any of the questions in the survey, or feel that you need support for your emotional well- being, you may find some useful resources here.

An important aspect of this study is to understand the later effects of the current pandemic on your physical and psychological well-being. We hope to evaluate this with two shorter follow-up surveys in the next 4 months. This will enrich the information collected from the study and hopefully guide further support to healthcare professionals in future pandemics. If you agree to be contacted, please provide your contact email below.

Please note that we will not ask you for any personal identifiable information and all data collected will be anonymized.

* **I** am happy to participate in the next phase of this study (NB: that survey will be considerably shorter)

- Yes
- No

CoPE-HCP Study

* My email address is:

CoPE-HCP Study

If you have any feedback, or comment on the CoPE-HCP study, please feel free to share it here.

If you need to contact us, please email us at cope.hcp@qmul.ac.uk

NB: please note that if you are concerned about your mental or physical health or any other health related issue, please contact your GP or occupational health departments. You may also like to use the online resources mentioned in our website

THANK YOU FOR PARTICIPATING IN THE CoPE-HCP STUDY!
